# CircMMP11 as a prognostic biomarker mediates miR-361-3p/HMGB1 axis to accelerate malignant progression of hepatocellular carcinoma

**DOI:** 10.1515/med-2023-0803

**Published:** 2023-11-02

**Authors:** Qiong Zou, Yuping Zhang, Daoqi Zhu, Xinrong Liu, Changfa Wang, Hong Xiang

**Affiliations:** Department of Pathology, Third Xiangya Hospital, Central South University, Changsha, Hunan, 410013, China; Department of The First General Surgery, Third Xiangya Hospital, Central South University, No. 138 Tongzipo Road Avenue, Changsha, Hunan, 410013, China; Department of Oncology, Changsha Kexin Cancer Hospital, Changsha, Hunan, 410205, China; Department of The First General Surgery, Third Xiangya Hospital, Central South University, Changsha, Hunan, 410013, China; Department of Central Lab, Third Xiangya Hospital, Central South University, Changsha, Hunan, 410013, China

**Keywords:** circRNA, invasion, hepatocellular carcinoma, migration, proliferation

## Abstract

As a high metastatic tumor, patients having hepatocellular carcinoma (HCC) show poor prognosis. The carcinogenic roles of circMMP11 are generally described in the development of other cancers. However, there is a lack of studies on its involvement in HCC. Therefore, we investigated the potential role and molecular mechanisms of CircMMP11 in the development of HCC *in vitro*, providing preliminary evidence for the clinical treatment of HCC. First, we examined the expression of CircMMP11 in HCC tissues and cell lines in both clinical and *in vitro* experiments. We then used a loss-of-function assay to determine CircMMP11’s regulatory role on the malignant characteristics of HCC cells. The results showed that high expression of CircMMP11 in HCC was associated with patient overall survival. Serum CircMMP11 had good diagnostic efficacy in distinguishing HCC patients from the control group. *In vitro*, inhibiting CircMMP11 suppressed the malignant characteristics of human HCC cell lines by directly sequestering miR-361-3p, which further affected the downstream gene HMGB1 expression. In addition, we knocked down CircMMP11 and found that its deletion inhibited the malignant characteristics of HCC cells through the miR-361-3p/HMGB1 axis.

## 1 Introduction

Although hepatectomy and liver transplant are acceptable treatment options for hepatocellular carcinoma (HCC) [1], early distant metastasis and malignant invasion remain the major obstacles to HCC treatment, leading to poor survival rate and unsatisfied prognosis [2,3]. In addition to traditional treatments for HCC, a study has found that traditional Chinese medicines can inhibit cell malignancy and epithelial–mesenchymal transition (EMT) progression in HCC and have been recognized for their multi-targeted and coordinated interventions in HCC [4]. Another study confirmed that immunotherapy improved the therapeutic effect of low dose sorafenib and avoided the adverse effects of chemotherapy [5]. However, further exploration of new therapies for HCC is still needed. To represent a promising diagnostic and prognostic biomarker in HCC, the concentration on an in-depth understanding of the molecular basis is extremely needed, such as tumorigenic and antitumorigenic roles of a novel class of endogenous noncoding RNA molecules, namely circular RNAs (circRNAs) [6].

Aberrant expression of miRNAs has been discovered in various diseases, such as neurodegenerative diseases, cancer, and inflammatory conditions [7–9]. Previous studies have found that miR-361-3p plays a promotive role in tumor metastasis and EMT processes in thyroid and pancreatic cancer [10,11]. In a recent study, the miR-361-3p/SOX4 axis was found to promote the development of HCC [12]. CircRNAs have the capacity of sponging miRNAs by competitively binding to the miRNA response elements with mRNA, thus affecting gene expression [13], which are implicated in diverse cancer types and regulated tumorigenesis positively and negatively [14]. CircMMP11 (hsa_circ_0062558) is a circRNA molecule that has been found to be expressed abnormally in breast cancer tissues [15–17]. It also regulates the expression of downstream genes by acting as a molecular sponge for specific microRNAs [17]. However, the role of CircMMP11 in HCC has not been researched yet. In our preliminary experiments, we found high expression of circMMP11 in HCC patients. So, we conducted this work to analyze its diagnostic and prognostic values in HCC patients. Furthermore, its potential role and molecular mechanism in the development of HCC were also estimated through the *in vitro* investigation, thus providing a preliminary clinical basis for HCC.

## 2 Materials and methods

### 2.1 Participants and sample collection

Peripheral whole blood (*n* = 40) collected in EDTA tubes, as well as the tumor (*n* = 60) and these corresponding adjacent normal tissues (*n* = 60) frozen in liquid nitrogen were obtained from HCC patients. In addition, 40 age- and sex-matched healthy subjects were also subjected to blood extraction. The baseline information of HCC patients is listed in Table 1.

**Table 1:** The clinicopathological features of HCC patients (n=60)

Characteristics	Total *n* = 60	High circMMP11, *n* = 30	low circMMP11, *n* = 30	*p*
**Age (years)**				
<65 years	17	10	7	0.156
≥65 years	43	20	23	
**Sex**				
Male	47	23	24	0.731
Female	13	7	6	
**Tumor size**				
<5 cm	51	24	27	0.073
≥5 cm	9	6	3	
**TNM stage**				
I–II	49	22	27	0.003
III–IV	11	8	3	
**Alpha-fetoprotein (AFP)**				
<400 ng/ml	14	6	8	0.371
≥400 ng/ml	46	24	22	
**HBsAg**				
Positive	54	27	27	1.000
Negative	6	3	3	


**Ethics approval and consent to participate:** The present study was approved by the ethic committee of Third Xiangya Hospital, Central South University.

### 2.2 CircInteractome predicted the binding site

CircInteractome is a web-based tool to explore the potential interactions of circRNAs with RBPs. This tool designs specific divergent primers to detect circRNAs, studies tissue- and cell-specific circRNAs, identifies gene-specific circRNAs, and explores potential miRNAs that interact with circRNAs. Our predictions with this tool show the binding site between circMMP11 and miR-361-3p. The URL of this tool is as follows: https://circinteractome.nia.nih.gov/.

### 2.3 Cell lines and cell transfection

Dulbecco’s Modified Eagle’s Medium (Life Technologies, Carlsbad, CA, USA) was used to culture the HCC cell lines, including Huh7, HepG2, HCCLM3, and Hep3B at 37°C in 5% CO_2_, as well as the immortalized human hepatocytes L02, which were obtained from American Type Culture Collection (ATCC, Manassas, VA, USA). HCC cell line Huh7 and Hep3B at 70–80% confluency were transfected with the following plasmids (Genepharma, Shanghai, China) using a commercial kit (Lipofectamine 2000, Invitrogen, USA), including si-circMMP11/si-NC (negative control/circMMP1 siRNA), pcDNA-based HMGB1 overexpression vector, pcDNA vector, and miR-361-3p mimic/inhibitor/NC (in-miR-361-3p: 5′-AAAUCAGAAUCACACCUGGGGGA-3, RiboBio, Guangzhou, China). Cells were cultured for 48 h before analysis.

### 2.4 RNA extraction and quantitative real-time PCR

Total RNA obtained via Trizol reagent (Invitrogen) was reversely transcribed into cDNA following the protocol of Reverse Transcription Reaction Kit (Takara), and the performance of PCR was done on a Real-Time PCR System (Applied Biosystems 7500). Table 2 lists the premier sequences. The relative circMMP11, miR-361-3p, and GAPDH expressions normalized to GAPDH/U6 were calculated using the CT method.

**Table 2:** Premier sequences

Gene		Sequences
circMMP11	Forward	5′-CTAGCTATGCCTACTTCCTGCG-3′
	Reverse	5′-CCAGAGCCTTCACCTTCACA-3′
miR-361-3p	Forward	5′-GCCGCTCCCCCAGGTGTGATT-3′
	Reverse	5′-GTGCAGGGTCCGAGGT-3′
PCNA	Forward	5′-TTGCACGTATATGCCGAGACC-3′
	Reverse	5′-GGTGAACAGGCTCATTCATCTCT-3′
MMP-2	Forward	5′-TGTCTTGCGTCTGACACTGC-3′
	Reverse	5′-CTCCTTTGGGCTAGGTATCTCT-3′
HMGB1	forward	5′-TATGGCAAAAGCGGACAAGG-3′
	Reverse	5′-CTTCGCAACATCACCAATGGA-3′
GAPDH	Forward	5′-TGTGGGCATCAATGGATTTGG-3′
	Reverse	5′-ACACCATGTATTCCGGGTCAAT-3′
U6	Forward	5′-AAAGCAAATCATCGGACGACC-3′
	Reverse	5′-GTACAACACATTGTTTCCTCGGA-3′

### 2.5 Cell viability detection using cell counting kit-8 (CCK-8) assay

The cell viability of HCC cell lines (Huh7 and Hep3B) at 0, 24, 48, and 72 h was assessed using CCK-8 detection kit (Sigma-Aldrich, St Louis, MO, USA) according to the user’s protocol using the OD value at 450 nm.

### 2.6 Assessment of migration and invasion using Transwell assays

The lower chamber contained 500 µL of culture medium with 10% FBS. Cells (1 × 10^4^) of HCC cell lines (Huh7 and Hep3B) were plated into 100 µL of serum-free medium in the upper chamber of the Transwell insert for migration assay, and a total of 5 × 10^4^ cells were loaded into a Matrigel-coated chamber instead for invasion assay. After 24 h, the migrated or invaded cells fixed and stained were counted under microscopy (200× magnification).

### 2.7 Western blotting

The protein isolated from cells were separated by electrophoresis and transferred onto the purchased membranes. After blocking, the membranes were hybridized with the primary and secondary antibodies (all from Cell Signaling Technology, Boston, MA, USA; 1:1,000 dilution), including HMGB1 (#6893), PCNA (#13110), MMP-2 (#40994), GAPDH (#5174, internal control), and IgG secondary antibodies labeled with horseradish peroxidase (#7074), followed by the detection using ECL reagent (Thermo Fisher Scientific, USA).

### 2.8 Luciferase reporter assay

HCC cells in 24-well plates at a density of 60% were transfected with the reporter construct containing wildtype (WT) or mutant of circMMP11 or HMGB1 and the miR-361-3p mimic/miR-NC using the transfection regent mentioned above. At 48 h post-transfection, the cells were collected for measuring the luciferase activities (Promega, Madison, WI, USA).

### 2.9 Biotinylated RNA pull-down assay

Using Pierce^™^ Magnetic RNA-Protein Pull-Down Kit (Thermo Fisher Scientific), the transfection of biotin-labeled miR-361-3p or miR-NC was performed in Huh7 and Hep3B cells. The whole-cell extract was incubated with streptavidin-labeled magnetic beads at 48 h post-transfection followed by isolation of co-precipitated RNAs and the detection using qRT-PCR.

### 2.10 Statistical analysis

All data were analyzed in SPSS (version 19.0, Inc, IL, USA) using *P* > 0.05 as not statistically significant via the following method, including Student’s *t*-test, one-way analysis of variance (ANOVA) followed by Tukey’s *post hoc* test, receiver operating characteristic (ROC) curve, Kaplan–Meier survival curves, and Pearson’s analysis.

## 3 Results

### 3.1 Expression of circMMP11 was significantly higher in HCC tissues and cells

**Figure 1:**
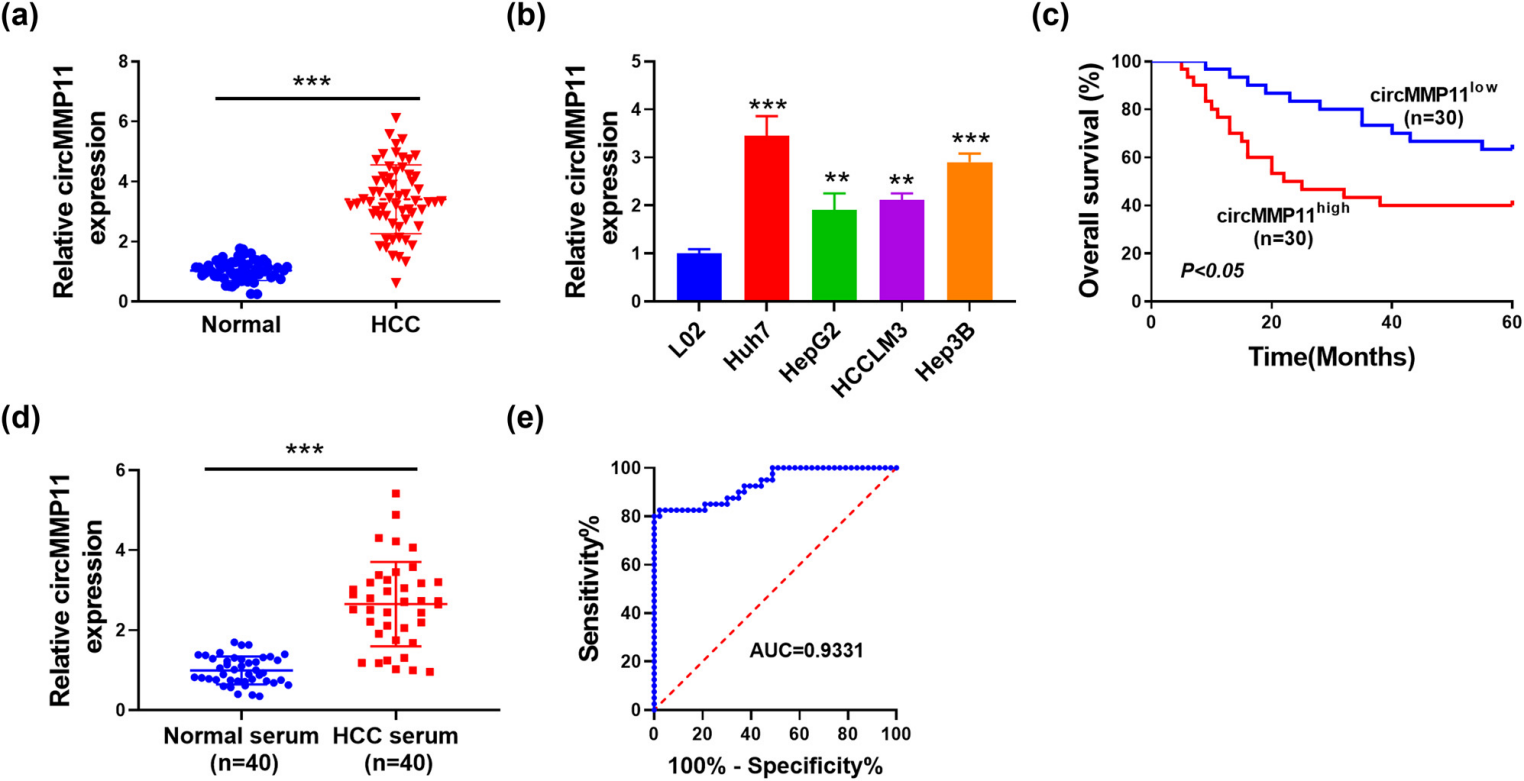
CircMMP11 is highly expressed in HCC. CircMMP11 expression in HCC and adjacent normal tissues (a), as well as in the indicated HCC or L02 cell line (b). (c) Kaplan–Meier analysis for HCC patients (*n* = 60) based on the expression of circMMP11. (d) Serum circMMP11 expression in 40 patients with HCC and 40 healthy subjects. (e) ROC curve for patients with HCC based on the serum levels of circMMP11. The continuous data were compared using Student’s *t*-test between two groups. **P < 0.01, ***P < 0.001.

As illustrated in Figure 1a and b, we used qPCR to measure the expression of circMMP11 in HCC tissues and cell lines and the results showed that higher expression of circMMP11 was observed in HCC tissues and cells than in the adjacent normal tissues and L02 cells, respectively. Additionally, the expression of circMMP11 in HCC cell lines was also determined. It was observed that compared with the L02 cell line, the expression of circMMP11 was significantly up-regulated in HCC cell lines (Figure 1b). Huh7 and Hep3B cell lines with the highest levels of circMMP11 were used for further experiments. According to the median value of circMMP11 in HCC tissues, patients with low expression exhibited a better prognosis compared to those with high expression (Figure 1c). Moreover, being consistent with the circMMP11 expression in tissue, its serum level was also enhanced in HCC patients as compared to the healthy individuals (Figure 1d), with the area under the curve of 0.9331 (Figure 1e), indicating that the circMMP11 expression was highly sensitive and specific for HCC.

### 3.2 Knockdown of circMMP11 inhibited malignant characteristics of HCC cells

**Figure 2:**
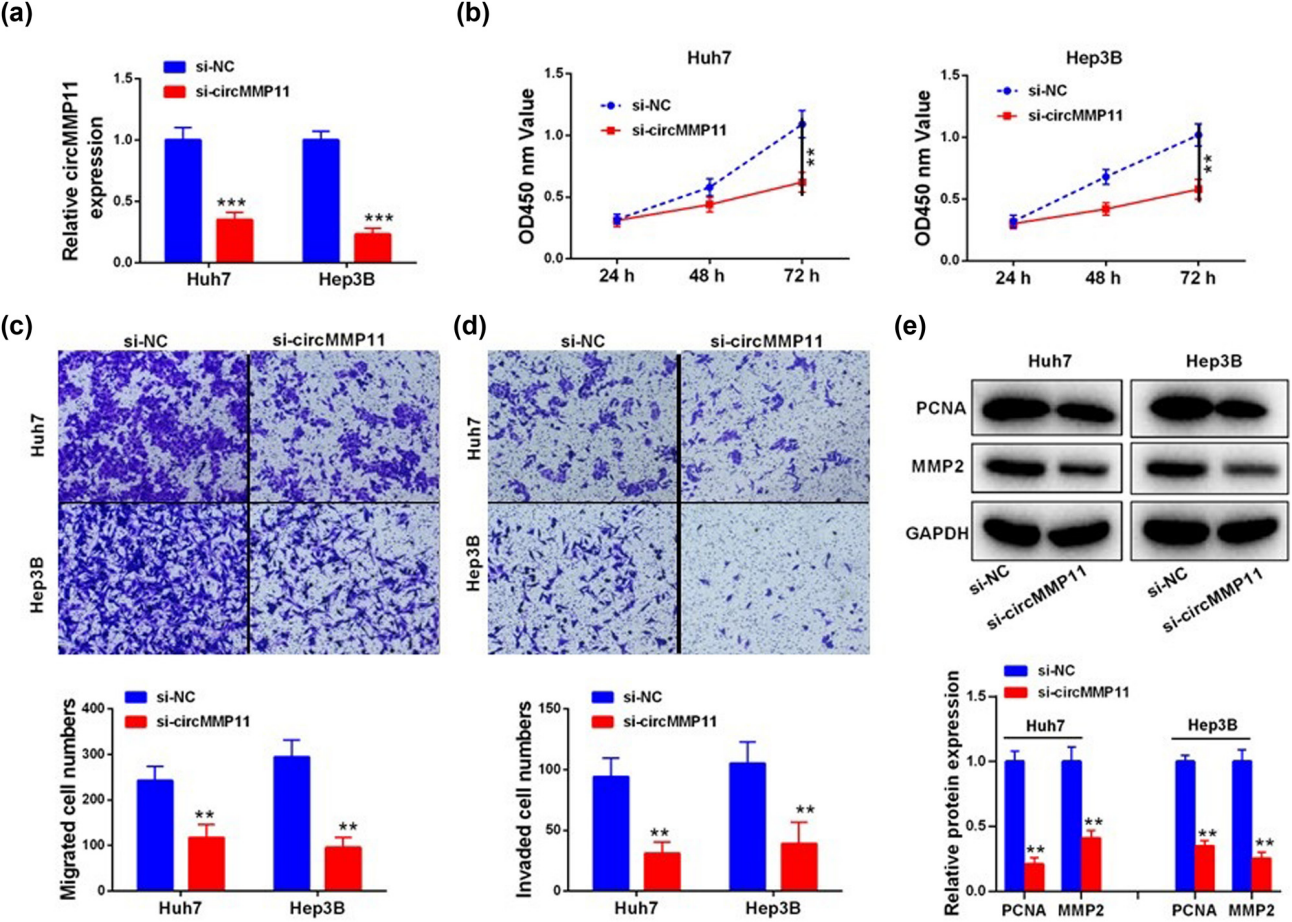
Knockdown of circMMP11 inhibits malignant characteristics of Huh7 and Hep3B cells. (a) Comparison of circMMP11 in HCC cells. (b–d) Comparison of malignant characteristics of transfected HCC cells, including the cell viability (b) and the number of migratory and invasive cells (c and d). (e) Comparison of PCNA and MMP-2 expression in HCC cells. Continuous data were compared using Student’s *t*-test between two groups. **P < 0.01, ***P < 0.001.

After using three siRNAs to silence circMMP11 expression in Huh7 and Hep3B cells (Figure 2a), we found that its knockdown inhibited the HCC cell viability (Figure 2b) with decreased numbers of migratory and invasive cells (Figure 2c and d), as well as the downregulated protein and mRNA levels of PCNA and MMP-2 (Figure 2e).

### 3.3 CircMMP11 interacted with miR-361-3p in HCC cells

**Figure 3:**
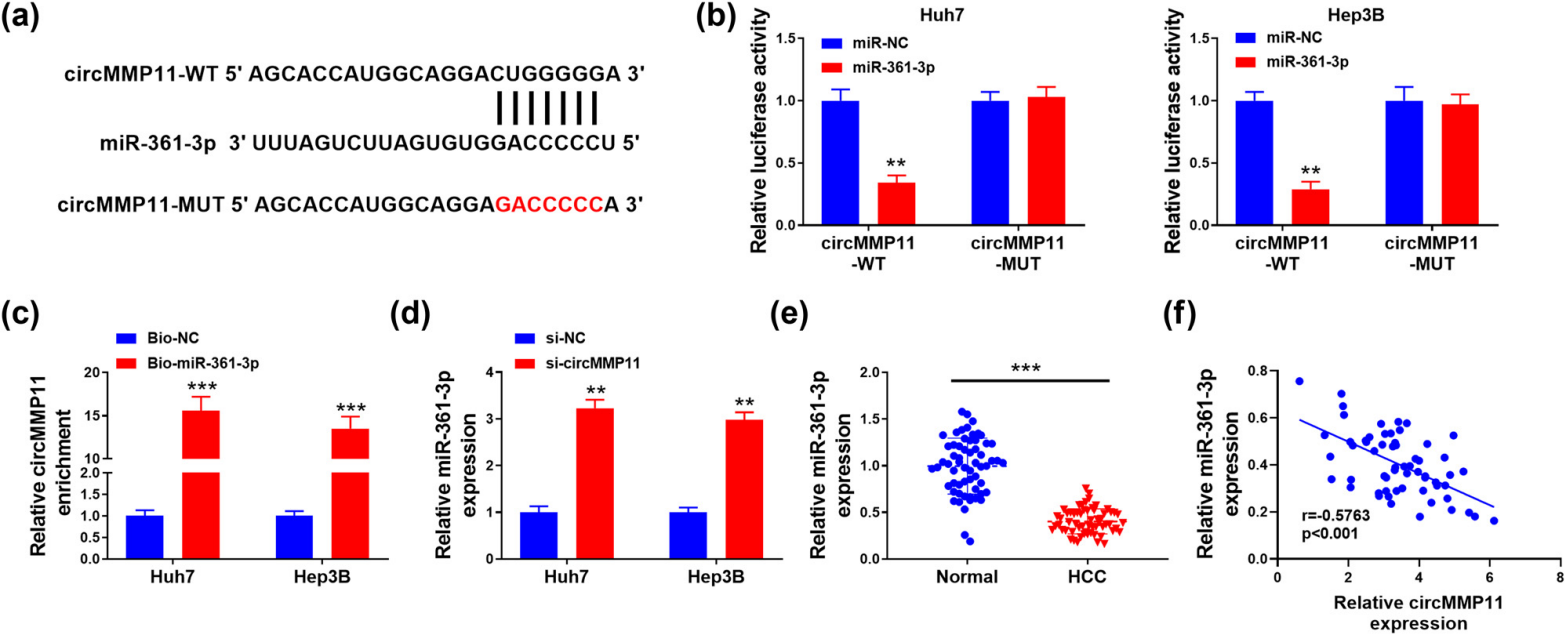
CircMMP11 in HCC cells interact with miR-361-3p. Putative target sequences between circMMP11 and miR-361-3p (a), and the validation of its relationship in Huh7 and Hep3B cells by luciferase reporter assay (b) and RNA pull down assay (c). (d) Expression of miR-361-3p in Huh7 and Hep3B cells were measured by PCR. (e) Expression of miR-361-3p in HCC and adjacent normal tissues were measured by PCR. (f) Relationship between circMMP11 and miR-361-3p in HCC. The continuous data were compared using Student’s *t*-test between two groups. Pearson’s analysis was used for correlation analysis. **P < 0.01, ***P < 0.001.

Bioinformatic prediction by CircInteractome showed the binding sites between circMMP11 and miR-361-3p (Figure 3a). Moreover, the relationship was validated in Huh7 and Hep3B cells via dual-luciferase assay (Figure 3b). Furthermore, the expression levels of circMMP11 exhibited more enrichment in the lysates of HCC cells transfected with the biotin-labeled miR-361-3p than those transfected with scrambled control (Figure 3c). In addition, miR-361-3p was significantly increased when circMMP11 was decreased in HCC cells (Figure 3d). As expected, miR-361-3p was significantly downregulated (Figure 3e), and inversely related to circMMP11 in HCC tissues (*P <* 0.001; *r* = −0.5763) (Figure 3f).

### 3.4 HMGB1 was a potential downstream gene directly regulated by miR-361-3p in HCC cells

**Figure 4:**
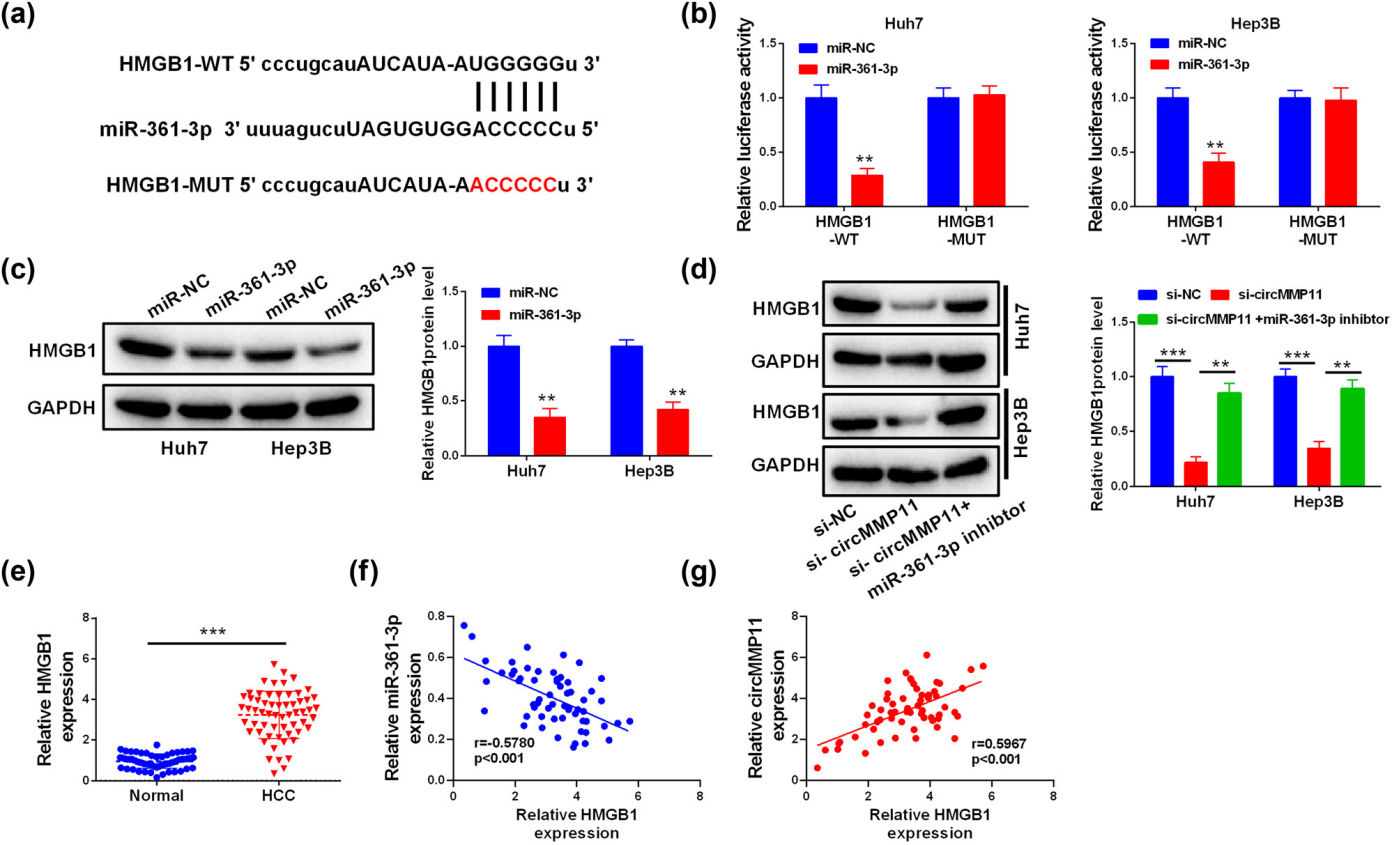
HMGB1 is a potential downstream gene directly regulated by miR-361-3p in HCC cells. (a and b) Schematic representation of the HMGB1 mRNA depicting miR-361-3p binding sites in 3′-UTR, and the validation in HCC cells by luciferase reporter assay (b). (c and d) Comparison of HMGB1 expression in HCC cells. (e) Comparison of the levels of MMP2 and HMGB1 in normal and HCC tissues (*n* = 60). (f and g) Relationships between HMGB1 and miR-361-3p/circMMP11 in HCC patients. Continuous data were compared using Student’s *t-*test between two groups. ANOVA followed by Tukey’s *post hoc* test was used for comparison among three or more groups. Pearson’s analysis was used for correlation analysis. **P < 0.01, ***P < 0.001.

Online prediction tool starBase revealed that HMGB1 is a predicted target gene of miR-361-3p (Figure 4a), and its mimics reduced the luciferase activity of HMGB1 WT in Huh7 and Hep3B cells (Figure 4b), and decreased the HMGB1 protein levels (Figure 4c). To validate the regulatory network of circMMP11-miR-361-3p-HMGB1 pathway, we examined the protein levels of HMGB1 in HCC cells after treating with miR-361-3p inhibitor (miR30017075-4-5, *in vivo*, 5 nmol, RiboBio, Guangzhou, China)/si-circMMP11 alone or in combination. As shown in Figure 4d, the decreased HMGB1 expression caused by the circMMP11 silencing was reversed by the inhibition of miR-361-3p. In clinical, upregulated HMGB1 mRNA levels were found in HCC tissues (Figure 4e), which were inversely correlated with miR-361-3p (Figure 4f) and positively correlated with circMMP11 (Figure 4g).

### 3.5 CircMMP11 exerted its oncogenic role in HCC cells via modulation of miR-361-3p/HMGB1 axis

**Figure 5:**
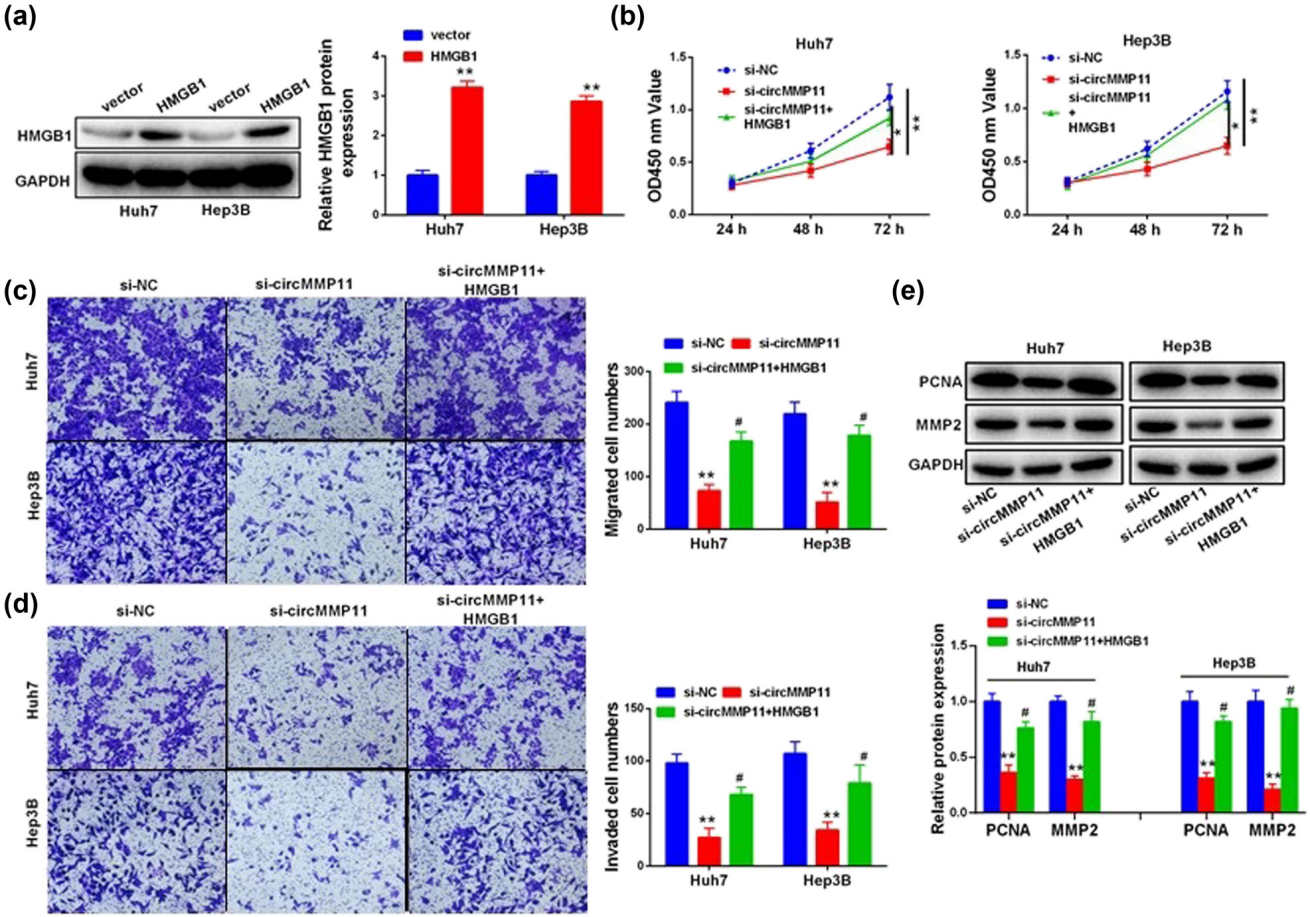
CircMMP11 exerts oncogenic roles in HCC cells via modulation of miR-361-3p/HMGB1 pathway. (a) Comparison of HMGB1 expression in HCC cells. (b–d) Comparison of malignant characteristics of transfected HCC cells, including the cell viability (b) and the number of migratory and invasive cells (c and d). (e) Comparison of PCNA and MMP-2 expression in Huh7 and Hep3B cells. Continuous data were compared using Student’s *t*-test between two groups. ANOVA followed by Tukey’s *post hoc* test was used for comparison among three or more groups. *P < 0.05, **P < 0.01, ^#^ *P <* 0.05.

The subsequent *in vitro* experiments were performed to determine whether circMMP11 exerted its biological function in HCC cells by regulating miR-361-3p/HMGB1 axis. As shown in Figure 5a, transfection with HMGB1 overexpression plasmid in Huh7 and Hep3B cells to increase the HMGB1 protein levels sufficiently reversed circMMP11 siRNA-induced inhibition of malignant characteristics of HCC cells (Figure 5b–d) and the levels of PCNA and MMP-2 (Figure 5e).

## 4 Discussion

First, the upregulation of circMMP11 expression in HCC tissues highlights its crucial role as a potential biomarker for diagnosis and prognosis. Second, further investigation supports the hypothesis that knockdown of circMMP11 inhibits the malignant characteristics of HCC cells by acting as a miRNA sponge for miR-361-3p through the inhibition of HMGB1, thereby providing a new therapeutic target for HCC. In recent years, several evidence suggested the diverse roles of circRNAs in numerous tumor biological processes [18,19]. For example, Wang et al. showed that circ_0004018 accelerated HCC progression by sponging miR-1197 to regulate PTEN/PI3K/AKT signaling pathway [20]. Zheng et al. indicated that circCCND1 directly sponges miR-497-5p and upregulates HMGA2 expression, leading to the tumorigenesis of HCC [21]. Recently, circMMP11 as molecular sponges for miR-1204, miR-625-5p, and miR-153-3p accelerated the development of breast cancer [15–17]. In this study, the HCC patients had upregulation of circMMP11 revealed in tumor tissues and were expected to have a poor prognosis. ROC analysis confirmed its high specificity and sensitivity, indicating that serum circMMP11 could be used as a diagnostic marker for HCC. Functionally, circMMP11 inhibition was capable of alleviating the malignant characteristics of HCC cells. As a molecular marker for proliferation, PCNA known in DNA replication [22] was increased in HCC tissues [23]. Moreover, another gene, MMP-2, which is produced by tumor cells, plays important roles in tumor invasion [24], including HCC [25]. Herein, PCNA and MMP-2 expressions in HCC cells were significantly decreased by circMMP11 knockdown. Collectively, we suggested that circMMP11 exhibited a tumor-promoting role in the development of HCC.

Notably, increasing studies show that circRNAs exert their functions through sponging miRNAs in various cancers, including HCC [26–28]. In our study, miR-361-3p, which was decreased in HCC patients [29], was found to be sponged by circMMP11, which was validated by corresponding experiments. Rescue experiments also verified that miR-361-3p knockdown attenuated the anti-tumor role of circMMP11 silence in HCC. Hence, we revealed that circMMP11 regulated the HCC progression by sponging miR-361-3p.

Furthermore, miR-361-3p could target HMGB1, which is an oncogenic molecule that has been implicated in various tumors [30–35]. High expression of HMGB1 produces the tumor-promoting role in HCC, which was capable of enhancing the growth and metastasis of HCC [36–38]. In some other studies on the interaction of HMGB1 with circRNAs, Cheng et al. demonstrated that circLPAR3 may play an oncogenic role by promoting HMGB1 expression through sponging miR-375/miR-433 [39]. Zhang et al. revealed that circ_0003645, a ceRNA for miR-139-3p, could upregulate HMGB1 and further cancer cell proliferation [40]. Yang et al. found that knockdown of circ-CSPP1 inhibited the development of HCC *in vitro* and *in vivo* by downregulating HMGB1, suggesting that HMGB1 and circ-CSPP1 are involved in the pathogenesis of HCC [38]. All these findings suggested that the mutual regulation of HMGB1 and circRNAs plays an important role in the development of tumor cells. Here, elevated HMGB1 expression in HCC tissues was inversely related to miR-361-3p but positively to circMMP11. Subsequently, the direct interaction between miR-361-3p and HMGB1 was verified. Interestingly, HMGB1 overexpression effectively alleviated the effect of inhibition of circMMP11 concerning the malignant behaviors of HCC cells by increasing the expression of PCNA and MMP-2. Together, the present study suggested that circMMP11 is capable of aggravating the malignant progression of HCC, which might be mediated by miR-361-3p/HMGB1 axis. However, the small sample size might result in the statistical limitation, and the targeted regulatory network of miR-361-3p in HCC needs to be explored.

In summary, circMMP11 shows promise as a diagnostic biomarker target and a valuable therapeutic target for HCC as it plays an anti-tumor role by suppressing proliferation, migration, and invasion in HCC, possibly through the miR-361-3p/HMGB1 axis. This work may provide a novel mechanism for understanding the progression of HCC.
